# A Treatise on Diseases of the Bones

**Published:** 1829-04-01

**Authors:** 


**A Treatise on Diseases of the Bones. By *Benjamin Bell*, Fellow of the Royal Colleges of Edinburgh and London. 12mo. pp. 291. Edinburgh and London, 1828.**

To the Student in surgery we know not a more puzzling and complicated task than the learning, or rather the understanding, of the various diseases of the osseous system. These difficulties depend in a great measure, no doubt, on the nature of the subject itself, but they certainly are increased, and the obscurum converted into the obscurius by the want of arrangement and barbarous jargon by which it is confounded. When Lord Tenterden was told, in the course of a recent and memorable trial in the Court of King's Bench, that cellular membrane, cellular tissue, cellular structure, and cellular substance, were synonymous and identical, he lifted up his hands in absolute dismay. We should like to observe that venerable judge, when some medical witness in trials "yet unborn," shall have to lay down the distinctions between caries, death, exfoliation, and necrosis of a bone, or descant upon osteo-malacia, spina ventosa, osteo-sarcoma, cystic sarcoma, osteo-steatoma, and a dozen other of the most villainously cacophanous names in all surgery. However, if merely the names were at fault the matter would deserve but little reprehension, for Shakspeare tells us that "a rose by any name would smell as sweet," and all must have observed that the harder and more *outré* a term in anatomical or surgical description, the more it is treasured and the better it is retained in the minds of young men. It is not with the names then we quarrel so much as with the vagueness of description, the jumbling of terms, and the constant use of one for the other, which perplex and mislead the hearer or the reader. The soft parts are liable to certain morbid actions or states, which we denominate ulceration—sloughing—gangrene. The terms are arbitrary, but, if always used with the same signification, the terms are good, because each exhibits to the mind's eye a peculiar condition of parts, as well and as truly as if it were actually presented to the material organ of vision. The bones too are subject, as well as the softer textures around them, to inflammation, ulceration, sloughing, &c. but here come the difficulty, for scarcely any two individuals describe these conditions alike. A patient has a sinus over his tibia, the surgeon introduces his probe, and he tells you, "with look profound," that the bone is dead, or it is carious, or both. Now, what are we to understand by *dead* bone? its sloughing or ulceration, for one of the two it must inevitably be? If we cannot employ the terms ulceration or sloughing in hard parts as well as in soft, for heaven's sake let us have names that we shall know when we see them, names that shall not denote one state to-day, and another state to-morrow, but words which are things, and speak a simple and intelligible language.

We merely allude to these circumstances at present, hereafter we shall notice particular points, and indulge in some remarks in the course of the following article. The work whose title we have placed at the head of this review, was conceived, as we are told by the intelligent author, in the hope of lessening those difficulties attending the study of diseases of the bones, which all must perceive, and which we have feebly endeavoured to portray. The writer who shall "reconcile the conflicting opinions which different authors entertain of the same disease," will execute an arduous but pleasing task, and deserve the thanks of the surgical part of the profession in general, of the student in particular. The following passage from the preface to the work explains its objects so briefly and yet so well, that we cannot do better than transcribe it.

"To satisfy myself respecting the nature of the various Diseases of the Bones, I embraced every opportunity of investigating them by the inspection of the human body, by experiments on the lower animals, and by the careful examination of morbid specimens, which are found in public and private collections. Thus prepared, I recorded for my own satisfaction, some observations on the pathology of bone; when it occurred to me, that, by publishing them in a condensed and systematic form, I might obviate some of the difficulties which occur to Students, in the investigation of this part of Morbid Anatomy. In doing this, it was not my intention to dwell upon the surgical treatment of the diseases which I notice: the brief remarks which I occasionally offer on the subject, may be considered as hints, to be followed out by the perusal of the works of respectable practical Authors.

"With a few exceptions I have described only those morbid conditions of the osseous tissue, which have come under my own observation. I have not, therefore, referred so frequently to the works of former writers as might have been expected. Besides, some of the information detailed in the following pages, was obtained in conversation with professional friends, and with this acknowledgment, I beg to offer them my sincere thanks; in particular to Sir ASTLEY COOPER, to Professors MONRO, BALLINGALL and TURNER, to Drs JOHN THOMSON and KNOX, to Messrs STANLEY, HOWSHIP, and A. SHAW, of London, and to Mr TURNER of Manchester." —*Preface.*

Mr. Bell has classed the diseases of bones under six general heads, each of them including a number of subdivisions. As this classification serves for the peg on which the observations of the author hang, we cannot pass it over here, and therefore subjoin it in his own words.

"I. The First Head includes the various kinds of Inflammation to which Bone and its Membranes are subject, viz.

"1. Inflammation of the Periosteum.
"2. Inflammation of the Surfaces of Bone.
"3. Inflammation of the Internal Structure, or Interstitial Inflammation of Bone.
"4. Suppurative Inflammation of Bone.
"5. Scrofulous Inflammation of Bone.
"6. Adhesive Inflammation of Bone.

"II. Under the Second Head are included the consequences of Inflammation, viz.

"1. Abscess in Bone.
"2. Ulceration of Bone.
"3. Mortification of Bone.

"III. The Third Head includes those affections of bone which apparently depend upon a morbid condition of its assimilating vessels

"1. Softening of Bone.
"2. Brittleness of Bone.
"3. Interstitial Absorption of Bone.
"4. Interstitial Deposition and Enlargement of Bone.
"5. Atrophy or Wasting of Bone.

"IV. The Fourth Head includes those preternatural growths from bone, which have not been proved to be the result of Inflammation, and which are not of a malignant nature.

"V. The Fifth Head embraces those incurable diseases which depend upon degeneration of the osseous tissue, viz.

"1. Spina Ventosa.

"2. Osteo-Sarcoma, or Fungus Hæmatodes of Bone.

"VI. A Sixth Head may with propriety include those anomalous affections of bone, concerning the nature of which little as yet is known.

"1. Bloody Tumour, or Aneurism of Bone.

"2. Tumours dependent upon the existence of Hydatids in the substance of the osseous tissue." 3.

The above arrangement is artificial, as most arrangements are, and faulty in many respects. What, for instance, can be more objectionable than the division into *separate* subdivisions, of inflammation, adhesive inflammation, suppurative inflammation, abscess in bone, and ulceration of bone? Inflammation is inflammation, and to divide it into species of adhesive and suppurative is absurd, because it is considering and naming it by its terminations, which terminations (abscess and ulceration) are afterwards treated of apart. We acknowledge, however, that in order to get forward at all in a work, it is absolutely necessary to adopt more divisions and subdivisions of the subject than nature perhaps would strictly warrant, and critics as we are, it has never been our practice, nor shall it be now, to cavil about trifles.

With regard to the affections and diseases of the periosteum which are first considered, Mr. B. remarks, that they consist in inflammation—morbid degeneration—and transformation of the tissue into a substance resembling bone. In the acute inflammation we find, on cutting through the tumour which is formed, that the periosteum is thickened, indurated, and separated from the bone by the interposition of a gelatinous or sero-purulent matter, whilst in the chronic there is merely thickening with increased density of the membrane. Mr. Bell is persuaded that many of those repeated cures of exostosis performed by empirics, by friction, &c. are nothing more than cases of indolent and chronic periostitis. The periosteum is subject to cancer, fungus hæmatodes, and most of the morbid degenerations which occur in common cellular texture, and we sometimes find that it loses its proper dense fibrous appearance, and becomes converted into a soft pulpy matter. After long continued chronic inflammation, the periosteum frequently acquires a cartilaginous hardness, and the earthy matter of bone is deposited. This deposite in time becomes united with the bone, and gives rise to a pseudo-exostosis, differing from the true in its lesser degree of hardness, and occasionally separated from the bone, from which it appears to spring, by a distinct line of demarcation. This ossified periosteum, having less vitality than exostosis, is now and then cured by the cautery, rubefacients, and so forth.

We now arrive at inflammation of the bone itself and its consequences, a part of the subject deserving much attention. Simple inflammation of the surface of the bone seldom exists uncombined with periostitis, and has never been observed by Mr. Bell on dissection; indeed, he believes that primary inflammation both of the periosteum and surfaces of bones is frequently confounded with inflammatory affections of the superjacent muscles, tendons, or ligaments. During life the acute form is marked by severe pain, referred to the bone, and increased upon pressure. If the soft parts are affected, there is generally swelling, usually of a simple inflammatory character, sometimes pitting upon pressure, but differing from common œdema in the violent pain produced upon pressure, as well as in the considerable constitutional irritation. Good illustrations of this disease are occasionally met with in cases of incipient paronychia, and in the joints of rheumatic subjects. In the chronic variety of the disease the appearances on dissection are tolerably well marked, for the periosteum is thickened, its vessels gorged with blood, and traceable on gently raising the membrane into the substance of the bone, especially the spongy bones, or the spongy extremities of the long bones. In some instances, this engorgement is so palpable as to lead one to suppose that it depended, in some measure, on a varicose state of the veins of the bone, and at times bloody points are observed on raising the periosteum, especially in the immediate vicinity of joints, or in parts of the bone which are covered by the synovial membrane.

The inflammation, above-described, of the superficies of a bone, may extend to its substance, constituting the interstitial inflammation, or this latter may occur, as the result of injuries inflicted on the bone, or, finally, as an idiopathic acute or chronic disease. When acute, it is extremely apt to run on to suppuration, but we sometimes find the distinctive phenomena sufficiently obvious. Besides the appearances which present themselves in superficial inflammation, the membrane which lines the cancelli is unusually vascular, or even thickened, and covered with a gelatino-fibrous matter; the coherence of the parietes of the cells is diminished, so that, on forcing the finger into the divided cylinder of a long bone thus inflamed, the gelatino-fibrous matter, mixed with blood, is squeezed out; the foramina for the nutrient vessels become enlarged, and considerable cavities are produced by the absorption of the bone, which should not be mistaken for caries, interstitial absorption, or fragilitas ossium; or, lastly, instead of absorption of the osseous tissue, gelatinous matter may be deposited in the cells, which, in process of time, becomes ossified, and renders the affected part more dense and compact in its structure than healthy bone. This thickening and consolidation has often been confounded with incipient necrosis, the bone being sometimes enlarged in its dimensions, and having exostotic productions on its surface, whilst its medullary cavity is filled with a spongy deposition of osseous matter.

The fourth section is occupied by the consideration of suppurative inflammation of bone, which our author considers as differing from abscess in bone, because "the formation of matter does not seem to be a *consequence* of inflammatory action, but a primary symptom, and one of its earliest phenomena." To this remark we cannot exactly subscribe, for we do believe that the pus which is formed is the sequence of inflammation, having, it is obvious, more of the erysipelatous than the phlegmonous character, and occurring in unhealthy individuals. As Mr. Bell observes, we frequently see it in the bones of stumps, which have been attacked with hospital gangrene, and then the ichoro-purulent matter may be seen oozing out from between the bone and periosteum, and, in some cases, from the cavity and cut surface of the bone itself.

"Suppurative inflammation affects both the surface and internal structure of bone. When it occurs superficially, the periosteum is separated or raised from the bone, a thin, ichoro-purulent discharge, or thick, unhealthy looking pus, covers its surface; and the external lamella is either of an ivory-white, or of a dirty grey hue: sometimes, too, small bloody points are seen on the surface of the bone. The purulent matter, or serous secretion, is not confined within any distinct sac or cyst, but is diffused between the periosteum and bone, over a considerable extent of surface.

In pure superficial suppurative inflammation, in general, no tendency to adhesive inflammation exists, but after it has affected the external lamina, it extends to the internal and cellular structure of bone.

"It is difficult, however, to determine in every case, whether the disease has commenced in the substance, or on the surface of the tissue. After the disease has existed for some time, the periosteum in general becomes inflamed and thickened, and sometimes ulcerated. When this takes place, the soft parts inflame, and collections of matter form in the cellular tissue between the muscles. These burst, and sinuses are the result. On introducing the probe into these sinuses, the bone can in general be found bare at the bottom of them; but this is not always the case, for occasionally the opening in the periosteum is not opposite to that on the surface, or is so small as not to admit the point of the instrument." 17.

We have seen, after acute rheumatic inflammation of the knee-joint and thigh, the periosteum stripped off the femur from condyle to trochanter, and the bone bathed in pus for the whole of that extent. Mr. Bell may call this "suppurative inflammation" if he chooses, but, in this case, undoubtedly inflammation *did* precede the formation of pus. We have witnessed two other most remarkable cases of extensive denudation and suppuration on the surface of the bone, one of which was a well-marked example of those purulent depositions following operations, and the other was a sequence of rheumatic fever.

The section on scrofulous inflammation of bone is very inferior to the excellent and accurate description of that disease, given by Mr. Brodie in his admirable work on Diseases of the Joints. One remark of Mr. Bell's is deserving of attention, namely, that scrofulous inflammation and ulceration of bone differs from caries (common caries or ulceration) in this, that it chiefly, if not entirely, implicates the fibro-cartilaginous structure, whilst caries attacks the whole substance of the bone, both animal and earthy. Scrofulous inflammation not unfrequently seizes on the bones of the cranium, and occasions the most extensive mischief. We witnessed a remarkable case of this kind, a year or two ago, at St. George's Hospital, and published the particulars at the time in this Journal. The disease appeared to have originated in the diploë of the right parietal bone, the whole of the outer table of which became dead, and the inner was very much destroyed. In spite of all this mischief, the patient suffered comparatively little; left the hospital of her own accord, to avoid any further surgical operation; and at present, we believe, is getting slowly well. We can bear testimony to the accuracy of our author's brief delineation of this scrofulous inflammation of the cranial bones.

"When scrofulous inflammation, attacks the bones of the cranium, it is in general preceded by inflammation of the cellular tissue, and periosteum covering them. A defined swelling, unattended by pain on pressure, is first felt; it is soft and elastic; and, in the course of a few weeks, if it is left to itself, an opening takes place, through which is discharged a sero-purulent matter, which has sometimes, though not always, a fetid smell. On introducing a probe into the opening, the bone is found to be bare, and sometimes rough on its surface. After some time, minute scales of the external lamina of the bone occasionally separate, and are discharged. If the disease stop here, granulations arise from the diploe, and an osseous cicatrix is gradually formed. But in individuals of a highly scrofulous diathesis, the disease penetrates completely through the bone. Even after this untoward event has taken place, and when the general health has been improved by the exhibition of appropriate remedies, a cure is, as I have sometimes seen, effected by means of the adhesive inflammation, and the solution of continuity is closed up by a deposition of a fibro-cartilaginous matter. That the destruction of the bone is not occasioned by the pressure of matter, but by what Van Helmont has termed *Corruptor*, and John Hunter *Morbid Action*, in the vessels of the bone itself, is proved by the fact, that a similar result ensues, when an early opening has been made, and the matter allowed to escape." 27.

At the same time it is proper, nay absolutely necessary, to give a free exit to matter which has formed, either by incisions through the scalp, or, if these are not sufficient, by trephining the outer table of the bone, when this can be easily reached. In considering the "adhesive inflammation of bone," Mr. Bell gives a very good account of the mode of union in simple and compound fractures. In the former, the soft parts around become swelled, from the rupture and distention of innumerable small vessels, together with the effusion of a quantity of serous fluid, mingled with blood, into the cellular tissue. In a short time after this effusion has taken place, a kind of gelatinous matter is secreted from the fractured extremities of the bone, which ultimately assumes a dense cartilaginous structure, and forms the nidus in which are deposited the earthy materials of the bone. If, however, the injury has been more extensive, portions of bone are killed and exfoliate, after which union takes place by a compound process. The surfaces from which the dead portions of bone have come away, are covered with minute florid granulations, that secrete a tough fluid, which, in process of time, assumes the appearance of fibro-cartilage, and is at length converted into bone. During the time that these offices are performed by the ends of the bone, whether in simple or compound fracture, the periosteum also is hard at work in forming an investing membrane for the callus. This new periosteum is always much thicker than the old for a considerable time, and, together with the unnatural size of the rough and unmodelled callus itself, as well as the inflammation and thickening of the soft parts, occasions that increase in bulk and consolidation, which are always felt over newly-united fractures. Passing over some remarks on non-union, we arrive at the second chapter of the work, dedicated to the consideration of the consequences of inflammation.

Abscess in bone is first considered, and a very important affection in practice it is. It is always, or almost always, the result of interstitial inflammation, and much more frequently met with in the shafts and epiphyses of the cylindrical bones, especially the tibia, than in any of the flat bones. It is not confined to the parietes of a bone, but occurs also in the medullary cavity, indeed the majority of the cases we have witnessed have been of the latter description. The abscess, which generally contains a sero-purulent fluid, is lined by a distinct membrane, and the neighbouring osseous tissue is filled with a gelatinous substance, like recent coagulable lymph, and occasionally somewhat enlarged. The absence of much disorganization distinguishes the common from the scrofulous abscess of bone, and the trifling enlargement from spina ventosa, with which, Mr. Bell assures us, it has often been confounded. The general symptoms produced by the formation of abscess in bone are frequently very severe, except in some cases of scrofulous disease of the joints, where their existence is scarcely known. Mr. Bell observes that, like other abscesses, these in bone have a tendency to make their way outwards, and, indeed, assumes as a general rule, that if left to themselves, the matter, in the greater proportion of cases, will work its way to the surface of the body. This may be as true as Holy Writ, but we do maintain that, if acted on in practice, the results will be most disastrous. None but those who have witnessed cases of abscess in the substance of the long bones can possibly appreciate the excessive constitutional disturbance they produce, and the almost immediate, the astonishing relief produced by applying the trephine, *puncturing* the abscess, and evacuating the matter thus "cabin'd, cribb'd, confined" in walls of bone. Mr. Brodie has lately directed the attention of the profession to these cases, and ably pointed out the symptoms of the disease, and the benefits of the operation of trephining, especially in the tibia.

Ulceration of the bone, or caries, comes next upon the *tapis*, and here we may observe that the term caries should be strictly limited to this signification. Carious bone and dead bone are certainly not synonymous terms, though often improperly used as such, for the latter means bone that has lost its vitality, and is either thrown off, or about to be thrown off, by the living bone, of which it before formed part and parcel. In the slow or chronic ulceration of bone, little can be noticed but what must be familiar to all; but another form of caries exists, "which resembles in its phenomena and progress the phagedenic ulcer of soft parts." The absorbents seem violently excited to action—no sloughs are thrown off from the substance of the bone—no granulations formed—but the sore increases in breadth and depth, occasionally penetrating through the walls of the bone, and producing an appearance not unlike the wound of a musket-ball.

"The phagedenic caries occurs most frequently in persons of an unhealthy habit of body, and in those who have a venereal taint. In syphilitic subjects the caries generally extends with great rapidity, both in a lateral and perpendicular direction; and in addition to its phagedenic characters, extensive sloughs or exfoliations are formed; there are no granulations, however, formed under the sloughs, but the diseased surfaces are highly vascular, and bleed when examined by the probe. When this disease attacks the flat bones, it very generally penetrates completely through their substance; but when it occurs in the long bones, it frequently confines its ravages to the external lamellæ. Good examples of this affection occurring as a purely idiopathic disease are to be met with in cases of that loathsome and intractable complaint of the nose and face, termed by most nosologists *Noli me tangere*.

"If, by means of proper treatment, the progress of the complaint be arrested, and the constitutional irritation which attends it subdued, nature, in many cases, makes powerful and successful efforts to repair the injury which the bones have sustained. Should the disease have confined its attacks to the external laminæ of the shafts of the cylindrical bones, healthy granulations are formed, the gelatinous matrix of bone is secreted, and osseous matter formed. There is one curious circumstance attending the new formation, which is worth remarking. Instead of presenting an uniformly dense texture throughout, there is interposed between the remaining lamellæ of the wall of the bone, and the external dense parietes of the new deposition, a kind of diploë, which, though more dense, resembles somewhat in structure the cancellar tissue of the bodies of the vertebræ. But if the caries has penetrated completely through the wall of the bone, and partially affected the meditullium, a dense osseous cellular deposite is formed, which fills up a portion of the entire cavity of the bone. The new osseous deposite is much more vascular than the original bone, and is hence liable to be easily excited, and to throw out exostotic excrescences." 43.

When this form of caries affects a spongy, vascular bone, like those of the tarsus and carpus, Mr. B. recommends its removal, more or less completely, by the knife. The disease requires energetic treatment, and, after having combated the constitutional disorder, the diseased portions of bone should be cut away as far as can conveniently be done, and the rest removed, by powerful escharotics, or the actual cautery, which stimulate, at the same time, to healthy action the vessels of the part.

The section on mortification of bone, or necrosis, is the next in order, and comprises a number of important points, and many very interesting remarks by the author. Necrosis may either affect the superficial laminæ, or entire tissue of part of a bone—the internal structure of the whole of a flat bone—or the internal laminæ and spongy texture of the cylindrical portion of a long bone. As a general rule, it may be said that external injuries of the cylinder of a long bone will produce necrosis; when inflicted on its spongy extremities—caries; a difference obviously depending on the greater degree of vascularity and vitality in the latter parts than in the former. Superficial necrosis may be either idiopathic or dependent on local causes, as external injury, or inordinate action of the muscles in unhealthy subjects. The idiopathic form is commonly the most extensive—generally prefers the flat to the cylindrical bones—and, in consequence of its being unattended with external wound, the exfoliations act as foreign bodies, occasion considerable irritation, and induce inflammation of the adjacent periosteum, suppuration in the soft parts around, and frequently troublesome caries of the subjacent bone. Although superficial necrosis is sometimes the result of inflammation originating in the soft parts and periosteum, Mr. Bell believes that caries will be found the most common sequela of such inflammation, and, on the other hand, that the death of the bone may at once be produced by a powerful external injury, or constitutional predisposition. So much for external necrosis.

"If we perforate a cylindrical bone, in one of the lower animals, by means of a sharp pointed instrument, and introduce any foreign body, such as a piece of lint or tow, into its cavity, we sometimes induce such a degree of inflammation in the medullary tissue, and in the internal layers of the interior parietes of the cylinder of the bone covering it, that death of the part takes place. The dead portion of bone, in the course of time, begins to act upon the more external layers of the bony parietes to which it adheres,—a high degree of inflammation takes place,—suppuration ensues,—and the necrosed portion is partially absorbed, and becomes enveloped in the matter which is poured into the medullary cavity of the bone. In process of time the inflammatory action extends from the cavity to the substance of the parietes of the bone; the bone gradually becomes interstitially enlarged throughout its whole circumference, and its highly stimulated vessels pour out new osseous matter on its outer surface and into the expanded cellular texture of the bone; while the absorbents, in the interior of the bone, being also excited, take up a considerable quantity of the decayed portion or sequestrum, which is shut up within. Many of the older surgeons imagined, and some surgeons of the present day are of opinion, that the reproduction of bone, after the separation of the sequestrum, in cases of internal necrosis, is dependent upon the periosteum." 54.

Our author moots the question of the reproductive powers of the periosteum in forming bone, but we regret that our limits will prevent our doing more than advert to it here; we therefore pass on to the pathology of that variety of necrosis where a portion of the entire cylinder of the bone is implicated. We witness this after bad gunshot wounds, compound fractures, or amputations, where the stump takes on an unhealthy action. The extremity of the bone in this latter case soon loses its vitality—the external lamina, for some distance above the point where it projects from the soft parts, also perishes, some of the internal laminæ and medullary tissue remaining uninjured, and throwing out granulations—the disease extends, perhaps, as high as the epiphysis—a large internal sequestrum forms—the external laminæ become affected with interstitial inflammation, deposition, and enlargement—and, at length, the bone presents all the phenomena and morbid appearances of internal necrosis. The most fortunate termination of necrosed stump is the union of the granulations from the central cavity with others which spring from the flesh of the stump, forming together a continuous cicatrized surface. The last kind of necrosis described by our author is that which results from progressive inflammation, or "progressive necrosis," a complicated and probably an idiopathic affection, occasionally called into action by an external cause.

"When, in consequence of a blow, or some other external injury, a portion of a bone—of a femur, for instance—is deprived of its vitality, a sequestrum forms, the outer tables of the bone become thickened, and exostotic productions form on the surface. The mischief does not terminate here, for while nature is endeavouring to repair the injury sustained in one part, another portion of the bone, in the vicinity of the original seat of the disease, becomes in its turn necrosed; and in this way I have seen the disease proceed progressively, until it has consumed nearly the whole of the original bone." 59.

In this affection two distinct processes, destruction and reparation, are going on at the same time, as in some of those compound sores of the skin, where we meet with phagedæna, suppuration, and granulation in combination. Mr. Bell concludes his observations on necrosis, by stating that they are founded on personal experience and experiment, and though he believes periosteum may become ossified in its texture, or at least impregnated with earthy matter, he still coincides in opinion with those who hold that the bone is regenerated by the vessels of its proper tissue. We have thus considered inflammation of bone and its consequences, suppuration, abscess, ulceration, and mortification, at some little length, because they are not very generally understood, are constantly occurring in practice, and are therefore matters of greater importance, than other more curious perhaps, but by far less common affections. The third chapter of this well-written little work, is on morbid affections of the bony structure, viz. softening, brittleness, interstitial absorption, interstitial enlargement, and, lastly, wasting of bone. The limits to which this review must of necessity be confined, will oblige us to select particular portions, rather than strictly analyse the remainder of the work.

We therefore pass over rickets and mollities ossium, which we cannot, by any means, agree with our author in considering identical, and stop for an instant at that curious affection, fragilitas ossium. A child, says Mr. Bell, falls upon the floor and fractures a limb, but the fracture is consolidated in less, perhaps, than the usual period. Some time afterwards, on lifting a moderate weight, or on giving the limb a slight twist, it is broken again, and again unites. This accident Mr. B. has seen occur three times in different parts of the right humerus of a child five years of age, within the short period of eighteen months. The patients that our author has seen have been healthy and seemingly free from scrofula, and in only two cases, was the structure of the bone apparently altered. In one, the muscles surrounding the humerus, the bone affected, were pulpy, the bone enveloped in partly fluid and partly coagulated blood, the bone itself friable, and perforated with small irregular shaped holes from its neck to the condyles, which reticular appearance was also observed in the osseous plates of the cancelli. Fragility of bones most commonly occurs in early and advanced life, constituting, in the former, a much less dangerous affection than in the latter.

The section which follows on interstitial absorption of bone is almost the longest in the book, and cannot be entirely neglected here. In the young, this affection is, in general, confined to the vertebral column; in the middle aged and elderly, its usual seat is in the neck of the thigh-bone, although Mr. Bell has seen it in other bones, and a specimen exists in the museum of the Edinburgh College of Surgeons, shewing it in the neck and head of the humerus.

The pathological phenomena presented are arranged by our author under two heads, the former comprehending the appearances presented in the periosteum, the latter those in the bone itself.

"I. The appearances observed in the investing membrane in some respects resemble those which are met with in simple superficial inflammation of bone, and in some varieties of exostosis. The periosteum is thickened, but there is no serous, purulent, or gelatinous deposit, interposed between it and the bone. It is vascular, but the vascularity does not resemble the uniform rosy blush of inflammation, and is more like congestion; the periosteo-osseous vessels are larger than they are found to be when the bones are in a perfectly healthy condition, and, on raising the periosteum, they may be distinctly observed to proceed from that membrane into the substance of the bone, penetrating, as it were, the external table, by an infinity of minute foramina.

"II. The bone, after maceration in water, seems to be, as it were, riddled by drills of different magnitudes. Its general texture, however, is not altered, the external laminæ and internal cellular substance retaining their proper degree of density.

"The foregoing appearances characterize the early stages of the affection, but when it has existed for some time, an evident loss of substance takes place in the bone, its opposite extremities approach nearer to each other; or when the absorption has taken place to a greater extent, upon one side or table of a bone, than upon another, the bone seems as if it were bent, and assumes a preternaturally concave appearance on one side, while the other side either retains its proper form, or presents a greater or less degree of convexity." 77.

The description of, and remarks on, interstitial absorption and lateral curvature of the bodies of the vertebræ are good in themselves, but not sufficiently novel to demand our particular attention. Mr. Bell hurls defiance at back-boards, stocks, and those "detestable contrivances of the mantua-maker and the dancing-master," bandages, braces, back-straps, steel-stays, and corsets, "engines" that are pregnant with dire, and often irremediable ill to the fair, of *both* sexes. Interstitial absorption of the neck of the thigh-bone is a matter, however, that is less understood by surgeons in general, indeed which is often mistaken for fracture. Such instances are daily occurring, and frequently occasion a lamentable loss of very useful breath in the members of our medical societies.

In persons advanced in life there is frequently an inclination of the body forwards, which, on accurate examination, is found to depend, not so much on a regular curve of the trunk, as a more or less acute one at the hip-joint, accompanied by another, comparatively slight, in the back and loins. This semiflexion of the pelvis on the femur is permanent—sometimes on one side only, when the limb affected is shorter than its fellow—the motions of abduction are limited—the patient cannot stand erect or use the least exertion without experiencing dull pain and a sense of weariness in the region of the hip-joint—rheumatic pains dart down the thigh, and spread across the loins—the muscles of the extremities waste—and the general health is in some instances impaired. Mr. Bell has seen examples of this disease in persons of thirteen, thirty, and forty years of age, but in these it did not appear to be an idiopathic affection, but rather the direct result of cold or mechanical injury. The appearances presented on dissection we must give in the words of the author, as they do not admit of curtailment.

"On reflecting the orbicular ligament, which invests and incloses the joint of the hip, we find the synovial membrane thickened, and in a highly vascular, though not inflamed, state. The thickening of this membrane is most apparent at that part where it begins to be reflected over the head of the thigh-bone at the edge of the cartilage. Occasionally the thickening exists in distinct portions of a round or nodulated form; but in the generality of examples it is diffused, and affects equally every part of the membrane. Occasionally, also, the capsular ligament is of an unusually dense texture.

"In addition to the thickening of the synovial membrane, those of its vessels which, in the healthy state, are colourless, appear very turgid, and of a bright red tint, anastomosing with each other, and giving rise to a beautiful reticulated appearance.

"In a more advanced stage of interstitial absorption, the periosteum, covering the distal portion of the neck of the femur, becomes thickened also, and injected with red blood. When this membrane is raised, the subjacent bone is found to be drilled, as it were, by an infinite number of minute holes, of calibres varying from the fifth of a line to a line in diameter. These holes, on minute examination, do not appear to penetrate deeper than the shell or external table of the bone, and are filled by the processes of periosteum which envelope the vessels of the osseous tissue. The foregoing phenomena characterize early stages of interstitial absorption, and are chiefly confined to the membranous parts. In the more advanced stages, we find, that, in addition to the thickening of the synovial membrane and periosteum, the bone is more thickly studded with the small foramina already alluded to, and these commonly exist in greatest number at the lower or concave surface of the cervix femoris. Something resembling a yielding or bending of the neck of the bone may now be perceived, for the inferior edge of the corona of the bone has approached to the lesser trochanter. This effect does not proceed from any softening or alteration in the intimate texture of the bone, but arises in consequence of the absorption and disappearance of a portion of its entire substance. On a cursory examination, it looks as if the head of the bone were forced downwards by the action of some great pressure; and I have seen cases in which the interstitial absorption had proceeded so far, that the head of the thigh-bone rested upon the upper part of the trochanter minor; and, in a specimen which is in my collection, that tuberosity is actually hollowed out, so as to afford a cavity for the head of the femur.

"It does not always happen, however, that the shortening proceeds to the greatest extent at the lower surface of the cervix, the upper surface being in some cases most materially affected; and there are specimens in pathological collections which prove that it may take place in every part of the circumference of the neck of the thigh-bone. In some rare cases I have observed, that, combined with the shortening of the cervix femoris, there is also a flattening of the head of the bone, and the formation of a deep groove around the lower edge of the corona. The absorption sometimes occupies even a wider range than has been mentioned above, more than two-thirds of the neck of the bone occasionally disappearing; so that the head of the bone is as if it were forced in the direction of its axis towards the base of the trochanter major." 95.

Frequently, besides the simple absorption, the lower part of the neck is incased, as it were, with a sheath of osseous, or rather exostotic depositions, cases that are especially apt to be confounded with fracture of the neck of the thigh-bone. The treatment recommended by our author consists in rest in the horizontal position—warm water poured upon the joint—counter-irritation by blisters, setons, issues, tartar-emetic ointment, moxas, the actual cautery, &c; but, considering that this, like ossification of the arteries or enlargement of the prostate, may in general be considered as one of those natural "woes," and not perhaps the worst, "that wait on age," we should hesitate before we had recourse to any very painful or severe applications. Two sections, one on interstitial deposition and enlargement of bone, and the other on atrophy or wasting, close the chapter on the morbid alterations of the osseous tissue. The atrophy or wasting, similar to what occurs in the softer textures, for the most part depends on the want of exercise, or, as Hunter would call it, the stimulus of action, and is met with in bed-ridden persons, or those who labour under diseases of the joints, which require in the treatment, almost absolute inaction in one or more limbs. We must also content ourselves with merely alluding to the section on exostosis, which, however, we recommend to the attentive perusal of the student, and pass to the subject of spina ventosa.

This disease has its general seat in the shafts of the long, and bodies of the short, cylindrical bones, and occurs in the young, the adult, or the aged.

"When a bone is affected with spina ventosa, its parietes seem, as it were, to swell out or expand; and hence, in its first stage, when no pain is felt by the individual in whom it occurs, as is frequently the case when it is seated in the metacarpal bones, it may be confounded with simple interstitial enlargement. As the disease advances, the expansion of the bone appears (if I may be allowed the expression) to be circumscribed, and to be confined either to the middle of the shaft, or to one of the extremities of the bone. The skin covering the diseased bone remains perfectly sound, and does not become ulcerated, nor discoloured, until a very advanced period of the ailment. The superficial veins, however, appear gorged, and form a kind of blue net-work on the surface of the swelling.

"As the tumour increases in bulk, the peculiar darting and burning pain, which attends its formation, becomes more and more severe, and if the limb in which it is situate be not amputated, acute irritative fever almost always supervenes.

"When the patient will not suffer an operation for his relief, the expansion of the bone proceeds to a prodigious extent, and by the distention of the soft parts which it occasions, at last produces ulceration of the attenuated muscular tissue and skin. From the ulcerated spots an unhealthy-looking ichorous pus is poured out, and the ulcerations degenerate into irritable sinuses, that closely resemble in appearance those which exist in the advanced stage of white swelling, or scrofulous inflammation of bone.

"On introducing a probe into one of the sinuses, it will be found that it communicates with an opening in the wall of the bone, and that the instrument may be pushed forwards, so as to traverse the diameter of the swelling." 128.

On examination of the bone after amputation, the periosteum is found in a thickened state, the shell of the expanded portion of bone remarkably attenuated, its internal surface lined by a delicate membrane, from which small spiculæ and plates of bone sometimes project, and the contents of the tumour, consisting of a sero-purulent matter, combined with a substance, in appearance, like gelatine.

"Spina ventosa differs from simple abscess of bone in the following respects:—1. Spina ventosa in general affects the apophyses, and seldom the epiphyses, of a bone; 2. Simple abscess, when it does occur in the shaft of a bone, is in general confined to one side of the wall of the bone, and while the pus contained in it is working its way to the surface of the body, organizable lymph, which at last becomes ossified, is poured into the medullary cavity and cancellar tissue, in the vicinity of the apostema. On the other hand, in the spina ventosa, the entire circumference and substance of a portion of the cylinder of the bone is affected, and no appearance of reparative or adhesive inflammation can be traced in the neighbouring cancelli or medullary canal.

"3. The comparatively trifling degree of swelling which attends common abscess, depends upon *interstitial enlargement and deposition* in the bone surrounding it, whereas the enormous tumefaction, which constitutes the peculiar characteristic of spina ventosa, is the result of *instertitial degeneration*, and the *actual expansion* of the attenuated walls of the bone, *at the seat*, and not in the vicinity, of the disease.

"4. Abscess of bone has been cured by means of the combined efforts of nature and art, without having recourse to amputation. Spina ventosa, so far as I know, has never been relieved, except by the removal of the *entire* bone in which it was seated.

"The treatment of spina ventosa is very simple, as the surgeon, when he is assured of its existence, must at once have recourse to the amputating knife. If the disease is seated in the bones of the metacarpus or metatarsus, as is generally the case in childhood, they should be removed at their articulations. If it has attacked the tibia and fibula, or radius and ulna, the amputation may be performed either at the knee or elbow, or a short way above these joints. The general rule to be observed is, that the entire bone, in which the disease has its seat, should be removed." 131.

The second section of the fifth chapter is occupied with the description of the malignant diseases of bone. These Mr. Bell arranges under two heads, the first consisting of cartilaginous degeneration—fleshy degeneration of bone—cystic sarcoma of bone—osteo steatoma—and encysted medullary sarcoma of bone, degenerations which are confined entirely to the osseous tissue itself, and never throw out fungous growths, nor ulcerate on their surfaces until a very late stage of their progress. Under the last head are considered cancer of the bones, fungus hæmatodes, and cartilaginous degeneration, diseases which implicate not only the bone itself, but also the periosteum and neighbouring soft parts, and have, when the skin is broken, a tendency to fungate. The whole are included under the generic term of osteo-sarcoma, which expressing an anatomical condition not present in many of the diseases it comprises, is highly objectionable. Mr. Bell himself admits the impropriety of the designation, but apologises for its use by his unwillingness to coin new terms. There was no necessity, however, for that, as the general heading, "malignant diseases of the bone," would have answered infinitely better than any barbarous Greek or Latin coinage. The diseases of the first class are so closely commingled with each other, that no rational hopes can be entertained of their ever admitting of accurate diagnosis in practice, indeed we believe that even the anatomical distinctions are much too finely spun. It is a comfort to think that a slip in diagnosis is no such great matter, for the treatment in all is the same—amputation, whenever it can be done without risk to the life of the patient.

The lines of demarcation between cancer and fungus hæmatodes are somewhat more broad, but alas, it can signify little to the patient whether his surgeon is aware that he is dying by one or by the other, for both are alike irremediable by art. Cancer of the bones, though not a common affection, is yet occasionally met with, and presents some well-marked characters. The disease, says Mr. Bell, in general depends upon extension of cancerous action from the neighbouring soft parts, and seems more especially confined to the periosteum and its interstitial processes. Those peculiar cartilaginous bands, so characteristic of scirrhus, proceed from the periosteum, which is thickened, and intersect the softened and disorganized bone. Between the cartilaginous intersections, the osseous tissue is converted into a soft matter, containing minute spiculæ, granules of bone, and cartilage. Ulceration at length takes place, the sore is covered by unhealthy, angry-looking, granulations, secreting a stinking ill-conditioned matter, and the patient dies a loathsome object. Cancer of the bone is rarely a primary affection.

"I know of no plan of treatment which is calculated either to cure or relieve this inveterate affection; the knife, gouge, and cautery, possess no power in arresting its progress; it extends its ravages from bone to cartilage and synovial membrane, and from them to bone again. The whole system appears to be contaminated; the stomach is irritable, and rejects its contents; the features are sunk, sallow, and listless; the eye anxious and dull; the skin harsh and dry; and the pulse rapid, thrilling, and feeble. All that the surgeon can do is, to attempt, by every means in his power, to allay the constitutional and local irritability, by the internal administration of hyosciamus, opium deprived of its narcotin, cicuta, digitalis, camphor, citrate of ammonia; and by the external application of the solution of chloride of soda, cicuta, and powder of the leaves of hyosciamus made into a paste with axunge, and applied to the diseased surface, beneath any common emollient cataplasm." 148.

Fungus hæmatodes Mr. Bell has never either seen or read of, as originating *in* the tissue of the bone. It invades it by extending from the neighbouring soft parts, and "seems to be produced in the bone, in consequence partly of the pressure, and partly of the altered condition of its nutrient vessels, which are involved in the diseased soft parts." We lately witnessed a well marked example of this disease, attached to the upper portion of the femur, but evidently not originating in the bone. The periosteum was in part destroyed, but whether in a primary or secondary manner is more than we can pretend to affirm. The "cartilaginous degeneration of bone, beginning in the soft tissue" is usually seen in the cavity of the antrum, and alveolar processes of the upper jaw.

"The first symptom of the disease frequently consists in the formation of a small and irregularly shaped indurated swelling of the gum. If this be not removed by the knife, the periosteum covering the alveolar margin of the superior maxillary bone becomes thickened, and of a dense structure; the bone beneath is at length affected, and is converted into a cartilaginous substance.

"It is frequently the case, however, that the disease arises, in the first instance, in the lining membrane of the maxillary antrum, and extends from it to the bony parietes. When this is the case, the earthy matter of the bone is absorbed, and the cartilaginous degeneration increases in bulk, and, if left to itself, gradually acquires a prodigious magnitude. It is surprising to remark the very great size that these malignant cartilaginous tumours attain, without causing any ulceration of the enormously distended integuments.

"Their formation and progress are sometimes unattended with pain, but in other cases they are productive of much local uneasiness and constitutional irritation.

"Cases have been met with, in which this disease has extended, previous to the occurrence of a fatal result, from the bones of the face to those of the cranium. It usually happens, however, that the bones below the orbit only are implicated." 152.

An operation for this disease Mr. Bell considers as not only useless, but worse than useless, and culpable even on the part of the operator. The sixth chapter, dedicated to anomalous affections of bones, and comprising the bloody tumour of bone, described by M. M. Breschet and Lallemand, and hydatid tumours of bone, can merely be glanced at here. Having given a full account of M. Breschet's memoir at the time that it appeared in the Répertoire, we shall not go over the ground again, although we may mention that there are two specimens of the disease in the Museum of St. Bartholomew's Hospital, one in that of the Royal College of Surgeons of Edinburgh, and one in Mr. Bell's collection. Those in the Bartholomew collection illustrate the disease as occurring in the humerus; in that which is in the museum of the College of Surgeons it affects the metarcarpal bones of the hand of a young woman, which was amputated by Mr. Joseph Bell; and the last mentioned instance is the humerus of a lady, whose arm was removed at the shoulder-joint, by Mr. G.

Bell, in the year 1823. This lady's case is detailed at length by Mr. Bell, and though we have not space for its insertion here, our readers will find the particulars in the periscope department of the Journal. The following is a summary of Mr. Bell's ideas on this curious disease.

"On considering attentively the phenomena presented in the cases described above, I am inclined to consider the bloody tumour of bone to be an affection similar in many respects to anastomotic aneurism of the other tissues;—like aneurism from anastomosis, it is sometimes, though not always, found to pulsate. It undoubtedly derives its origin from a morbidly increased development of the capillary vessels of the bone, conjoined most probably with increased vascular action of the second and third classes of arteries. It differs, however, from aneurism from anastomosis in other textures,—in this respect, that the structure of the bone, in some cases, undergoes alteration, and assumes a fibrous character; and in so doing, perhaps exhibits the power which nature possesses in accommodating parts to the changes which morbid action occasions. For, if the fibrous change of structure did not occur, a complete solution of continuity would be apt to take place, either from the tension occasioned by the flow of blood into the cavity of the bone, or from the mere contractions of the muscles of the limb.

"In those specimens of the disease which have come within the sphere of my observation, the parietes of the tumour have differed in appearance from those described by the French pathologists. In the cases mentioned by M. Breschet, the parietes were thin, and in some parts of a cartilaginous character; in the four preparations alluded to by myself, they consisted, partly of a thick and dense fibrous matter, and partly of bone." 163.

Of hydatids in the bony tissue our author has never seen an instance, and only read of three. M. Cruveilhier, in his first volume of the Anatomie Pathologique, page 236, relates a case from the hospital practice of M. Cullerier, where a number of hydatids, of the species termed by M. Laennec "*acephalocystes*," were contained in a tumour, situated in the anterior part of the tibia; Sir Astley Cooper, in Part I. page 163, of his Surgical Essays, has alluded to a case which occurred in the practice of Mr. Foster, at Guy's Hospital, where the hydatids were also found in the upper part of the tibia; and, lastly, Mr. Keate, in a very valuable paper in the tenth volume of the Medico-Chirurgical Transactions, has described the case of a young woman, where a tumour, externally resembling exostosis, was seated in the frontal bone, which, on being cut into, disclosed hydatids, surrounded by a serous fluid. These cases comprise the whole of what is known upon the subject, which we need not say is lamentably little. In the treatment of the affection, Mr. Bell recommends us to strike at once at the root of the disease, and endeavour, in the first instance, to remove as much of the membranous parietes of the cavity, containing the hydatids, as the safety of the patient will permit. Having done this, the sides of the cavity should be powerfully cauterized, and that as often as a single hydatid cyst remains, however minute it may be.

Our work of analysis is now at an end; for the remainder of the volume, consisting of upwards of a hundred pages, does not fall within the scope of a review. The observations on fractures of the neck of the thigh-bone are ingenious and just, but the subject has of late been so hacknied, in every imaginable shape, that we would not for the world inflict it again upon our readers. It is usual for the critic, on parting with his author, to pronounce a sort of judgment on his work, and deliver his *novissima verba*, whether of reproof or approbation. The lengthened notice we have taken of this little book of Mr. Bell's is the highest praise that we can offer, the most flattering earnest of our sense of its merits. It is really worth more than half of those tumid octavos and quartos, pampered, by the care of their authors and printers, into an unwieldy and unwholesome obesity. We conscientiously recommend it, not only to those students to whom it is so modestly addressed, but also to our surgical brethren in general.

